# Impact of COVID-19 on the utilisation of maternal health services in Bangladesh: A division-level analysis

**DOI:** 10.7189/jogh.14.05040

**Published:** 2024-11-22

**Authors:** Aniqa Tasnim Hossain, Ema Akter, Ridwana Maher Manna, Tasnu Ara, Md. Alamgir Hossain, KM Tanvir, Md Hafizur Rahman, Abu Sayeed, Abu Bakkar Siddique, Bibek Ahamed, M Sabbir Haider, Sabrina Jabeen, Shafiqul Ameen, Mohammad Sohel Shomik, Anisuddin Ahmed, Luis Huicho, Alicia Matijasevich, Abdoulaye Maiga, Ahmed Ehsanur Rahman, Nadia Akseer, Shams El Arifeen, Agbessi Amouzou

**Affiliations:** 1 Maternal and Child Health Division, International Centre for Diarrhoeal Disease Research, Dhaka, Bangladesh; 2 Institute of Statistical Research & Training, Dhaka University, Dhaka, Bangladesh; 3 Institute of Epidemiology, Disease Control and Research, Dhaka, Bangladesh; 4 Nutrition and Clinical Services Division, International Centre for Diarrhoeal Disease Research, Dhaka, Bangladesh; 5 Centro de Investigación en Salud Materna e Infantil, Centro de Investigación para el Desarrollo Integral y Sostenible and Facultad de Medicina, Universidad Peruana Cayetano Heredia, Lima, Peru; 6 Departamento de Medicina Preventiva, Faculdade de Medicina FMUSP, Universidade de São Paulo, São Paulo, Brasil; 7 Department of International Health, Johns Hopkins University Bloomberg School of Public Health, Baltimore, Maryland, USA

## Abstract

**Background:**

The coronavirus disease 2019 (COVID-19) pandemic had substantially disrupted maternal health care provision and utilisation in Bangladesh. However, the extent of geographical disparities in service utilisation and how the health system withstood these challenges have not been studied. This study explores the divisional disparities in trends and disruptions in maternal health service utilisation caused by the COVID-19 pandemic.

**Methods:**

Data was extracted from the District Health Information Software of Bangladesh from January 2017 to December 2021. We assessed the trend of first antenatal care visit, institutional delivery and number of caesarean sections over these years. We explored both the yearly and monthly trends to see the variations in the number of utilisations. Segmented regression with Poisson distribution was used to assess changes in service utilisation during the COVID-19 period. We reported incidence rate ratio (IRR) of service utilisation with a 95% confidence interval (CI) in different divisions during COVID-19 (2020–2021) compared to the reference period (2017–2019).

**Results:**

Initially, a notable decline in maternal health care utilisation was observed in 2020 compared to the pre-pandemic period of 2017–2019. Divisional disparities were observed in this trend. Overall, compared to the pre-pandemic period, we observed around 30% decline in all three selected indicators of maternal health care. The lowest value was observed in Chattogram in 2020 (IRR = 0.66; 95% CI = 0.55–0.79) and Rajshahi in 2021 (IRR = 0.71; 95% CI = 0.60–0.82). For institutional delivery, Barishal division had the lowest IRR (0.64; 95% CI = 0.60–0.68) in 2020 and, in 2021 Rajshahi had the lowest IRR (0.71; 95% CI = 0.60–0.82). For caesarean section, the lowest value was observed in Barishal division (IRR = 0.48; 95% CI = 0.44–0.53) in 2020 and in Mymensingh (IRR = 0.37; 95% CI = 0.32–0.43) in 2021. By 2021, the three maternal health care utilisation indicators demonstrated recovery.

**Conclusions:**

The effect of the pandemic, including lockdown, on the selected maternal service utilisation was observed in Bangladesh though there were substantial geographic disparities. These disruptions slightly recovered after the initial shock. These results will support the government in preparing the national and regional health systems for future epidemics in Bangladesh.

Coronavirus disease 2019 (COVID-19) had a profound impact on people’s lives globally [1]. Around 700 000 000 cases were identified and 7 000 000 deaths were recorded directly attributed to COVID-19 [2]. Like other countries, Bangladesh also experienced devastating impacts of the pandemic with around 29 000 officially reported deaths [2]. However, these numbers are likely underreported because of the lack of a strong surveillance system to detect all COVID-19-related cases and deaths. [3]. The World Health Organization (WHO) reported 141 000 people died as a result of COVID-19 in Bangladesh between 2020 and 2021 which is much higher than the official records [4]. Globally, the pandemic and lockdown that were imposed as control measures have been reported to have disrupted essential health services [5].

The government of Bangladesh announced a nationwide lockdown to prevent the disease spread from 23 March to 30 May 2020 [6]. This lockdown-imposed restrictions on the international and local travel, commercial activities, activities under educational institutes and social gatherings. Social distancing and limited travel restrictions continued to prevent further circulation of the disease. During the second wave (after the last week of March 2021) of COVID-19 in Bangladesh, the government announced another national lockdown with international travel restrictions in April 2021 [7,8].

Although restrictions were imposed on travels and social gatherings to maintain the social distancing, people were allowed to access all essential health services during lockdown periods. Yet during the pandemic period, 11% of pregnant women received antenatal care visit (ANC) in their third trimester, while 22% received it in pre-pandemic period [9]. Among other reasons, service disruption occurred due to the shortage of physician and nurses, since they were busy with COVID-19 efforts [10,11]. Besides on COVID-19-specific planning and reactions, multiple efforts have been initiated to increase the availability and readiness for essential maternal and neonatal health services such as guideline development, organised virtual meetings, recruitment of physicians and nurses [9,12,13].

Despite these efforts on the national level, the impact of COVID-19 on health care utilisation on divisional level has not been extensively investigated or documented in Bangladesh. Although the lockdown was nationwide, the response to health care systems may have been different in terms of accessing care as the case detection in different division varied widely [14]. A cross-sectional household survey conducted in a rural subdistrict reported the indirect effects of the early phase of the COVID-19 pandemic on the coverage of essential maternal health services [9]. This study reported that the effect of the early phase of the pandemic including lockdown on the selected maternal and child health service coverage was null in the study area. This needs further investigation at the subnational level to assess the impact of COVID-19 on the service use. The majority of the studies reported changes on a national level. Evidence on division level is still unavailable in Bangladesh. Also, most of the available evidence showed impact during the first wave of COVID-19. It is also important to document utilisation of routine health care services under different waves of the pandemic in Bangladesh.

National Health Management Information System (HMIS) of Bangladesh gives us a good opportunity to explore the trend and pattern of utilisation of health care at the public facilities over time [15,16]. Bangladesh health management information system adopted the District Health Information Software, version 2 (DHIS2) to capture updated health service utilisation data in 2009. Our study aims to explore the indirect effects of COVID-19 on maternal health indicators using routine health information data by different geographical divisions in Bangladesh. This study will support a responsive health system approach at the division level to prepare for the future epidemics in Bangladesh.

## METHODS

### Study settings

**Figure 1:**
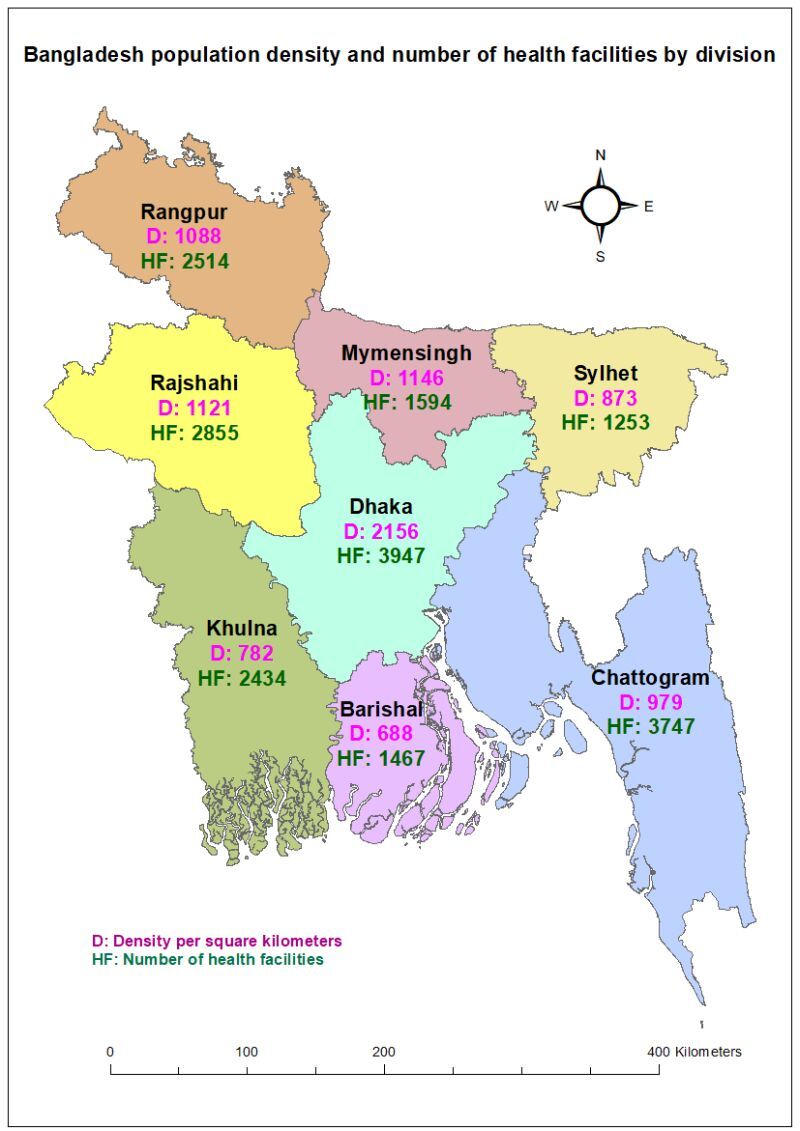
Population density and number of health facilities by division.

This study used information of the public facilities of Bangladesh extracted from DHIS2. The People’s Republic of Bangladesh is a country in South Asia with GDP *per capita* of 2688 USD (in 2022), and a land area of 147 547 km^2^ [17,18]. The country has around 165 million population and is one of the most densely populated countries in the world (1119 people per square kilometre) [19]. Administratively, the country is divided into eight divisions (**Figure 1**). We have included the divisional information in this study. The divisions are Barishal, Chattogram, Dhaka, Khulna, Rajshahi, Rangpur, Mymensingh and Sylhet.

The health service in Bangladesh is governed by the Ministry of Health and Family Welfare [20]. District Health Information Software, version 2 (DHIS2) operates under the health services division governed by the Ministry of Health and Family Welfare [21].

### Data sources

The data on monthly counts of service utilisation from January 2017 to December 2021 on division level was extracted from DHIS2 Bangladesh. It provides data on health-related indicators for routine monitoring and evaluation at the facilities. The data covers mostly public facilities because most of the private facilities do not report maternal health care service utilisation [16,22,23]. Data was collected on a monthly basis from all public facilities in all eight divisions in Bangladesh, namely Dhaka, Chattogram, Barishal, Khulna, Rajshahi, Rangpur, Mymensingh and Sylhet.

### Outcome variables

The main outcome variables are number of pregnant women who had at least one ANC visit, number of institutional deliveries and number of women delivering through caesarean section. These variables are mostly used for monitoring the maternal health care utilisation.

### Assessment and adjustment for incomplete reporting

We verified the completeness of the reporting for our selected indicators using supplementary data from DHIS2. Our adjustment method was based on assumptions about the levels of service outputs (pregnancy care and deliveries) at times when data was not reported, in comparison to periods with complete reports.

We employed the following formula to adjust for incomplete reporting:

N (adjusted) = N (reported) + N (reported) × (1 / (c) - 1) × k

where N represents the number of service outputs, c is the reporting completeness, and k is an adjustment factor. The adjustment factor k takes on values representing the assumed level of services in non-reporting time points, ranging from 0 (indicating no services) to 1.0 (indicating the same rate of services as reporting period). The values of k are as follows:

k = 0; no services during non-reported time point

k = 0.25; some services, but much lower than reporting time point

k = 0.5; half the rate compared to reporting time point

k = 0.75; nearly as much as reporting time point

k = 1.0; same rate of services as reporting time point.

The choice of the adjustment factor (k) requires consultation with experts with knowledge of the country’s health system. We have used the k = 0.75 as per the expert suggestion of Bangladesh, where it is assumed that we have some variation of service provision between the reporting and non-reporting facilities. Additional information on DHIS2 data quality is provided in Appendix S1 in the **Online Supplementary Document**.

### Statistical analysis

We analysed the yearly trend to ascertain the increase or decrease in health care utilisation during the COVID-19 period. Next, we assessed the monthly trends to understand which time periods were mostly affected by COVID-19. We used segmented Poisson regression to quantify the size of any disruption in the trends in the monthly utilisation numbers. We have assessed the normality of the outcome variables and they were not normal which is an assumption for linear regression. Therefore, we fitted the Poisson regression which is widely accepted in such analyses for interrupted time series. The incidence rate ratios (IRR) are reported for explaining the impact of COVID-19 (2020, 2021) on our selected outcome variables compared to pre-COVID time (2017–2019). We have checked autocorrelation using Durbin Watson statistic [24]. We have also assessed our data for seasonality using Dickey-Fuller unit-root test and controlled for seasonality in the model [25]. Additional details on seasonality adjustment are provided in Appendix S2 in the **Online Supplementary Document**.

We have selected segmented Poisson regression with following justifications. Hospital service utilisation data, such as the number of visits or procedures, are count data. Poisson regression is specifically designed for modelling count data, making it a suitable choice. Segmented Poisson regression can model rates that change over time, which is essential for capturing the dynamic impact of COVID-19 as the situation evolves. It allows for the analysis of interruptions and interventions, such as lockdowns or policy changes, which have significant effects on service utilisation patterns. This method offers flexibility in modelling and understanding trends and patterns before, during, and after the COVID-19 pandemic periods.

We have quantified the rate of change by month during the pandemic period compared to pre-pandemic along with number of deaths per month. We reported the findings on divisional level. We used Stata version 15 (Texas, USA, 2015) for the quantitative analysis [26].

## RESULTS

**Figure 2:**
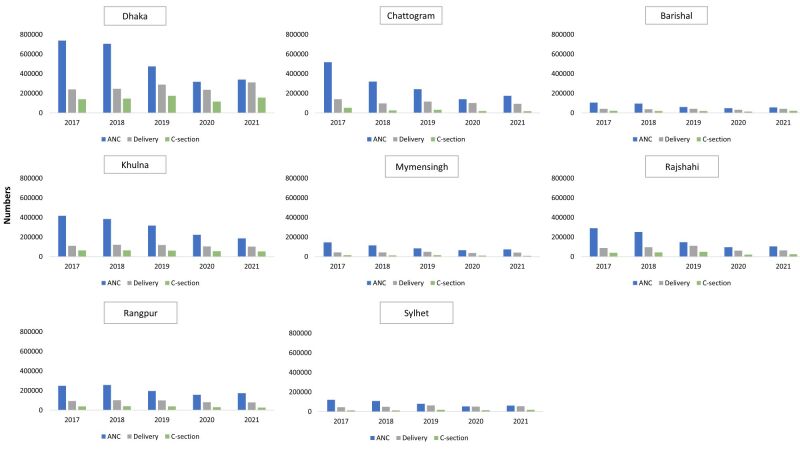
Yearly trend of maternal health indicators, number of first Antenatal Care contact, number of institutional delivery and number of caesarean sections from DHIS2 by divisions in Bangladesh. DHIS2 – District Health Information Software, version 2.

We present the annual changes in the number of ANC first visit, institutional delivery and caesarean section over the five-year period from 2017 to 2021 on divisional level (**Figure 2**).

### ANC first visit

There was a decline in ANC first visit from 2017 to 2019 in all divisions, with a dip in 2020 followed by a rebound in 2021. In all regions, the number of ANC services was highest in 2017, followed by a gradual decrease in subsequent years. Dhaka, despite having the highest absolute numbers of ANC services each year, saw a dramatic drop from around 0.74 million in 2017 to 0.32 million in 2020, indicating Dhaka was heavily impacted by the pandemic. In contrast, regions like Rangpur experienced less dramatic declines. For instance, Rangpur's ANC services dropped from 0.25 million in 2017 to 0.16 million in 2020 and slightly increased to 0.17 million in 2021, indicating a relatively more stable trend compared to Dhaka. In 2021, all the divisions experienced a substantial rebound but it is comparatively lower than the previous years of 2017–2019.

### Institutional delivery

The graphs suggest a steady upward trend in the number of institutional deliveries from 2017 to 2019 in all divisions, with the exception in Chattogram. During the 2017–2019 period, Chattogram experienced a decline in 2018 compared to 2017, followed by an increase in 2019. The number of institutional deliveries in Chattogram in 2019 remained lower than the level observed in 2017. In 2021, the divisions experienced a substantial rebound except Chattogram, Khulna and Rangpur.

### Caesarean section

A gradual increase in the number of caesarean section deliveries was observed from 2017 to 2019 in all divisions, except for Chattogram, which showed a decrease, and Barishal, where a decline was recorded in 2018 followed by consistency in 2019. In 2021, Dhaka, Barishal, Rajshahi and Sylhet showed a rebound in the trend from 2020 to 2021. While Chattogram, Khulna, Mymensingh and Rangpur reflected a drop in the numbers during 2021 as compared to 2020.

### Monthly change: ANC first visit

**Figure 3:**
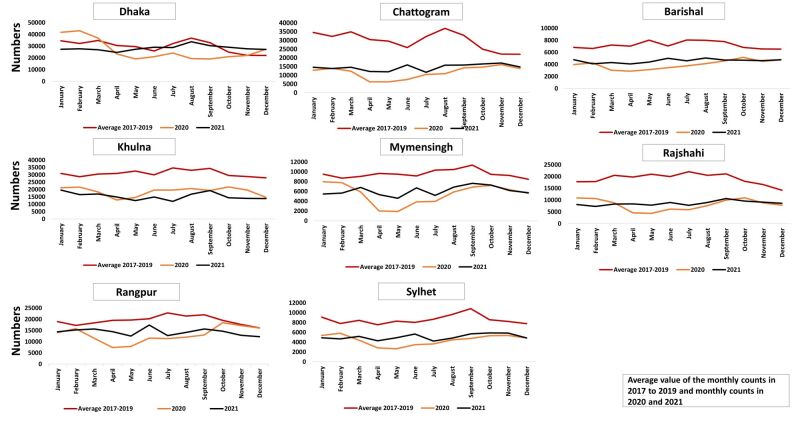
Monthly trend of first antenatal care visit using DHIS2 data set from 2017 to 2021. DHIS2 – District Health Information Software, version 2.

In the year 2020 in all divisions, there was a noticeable drop in ANC visits in April, followed by a gradual increase in subsequent months. The highest decline in April 2020 was observed in Mymensingh. The number of ANC visits in 2021 was higher than in 2020 but never reached the levels observed in 2017–2019 in all divisions, except Dhaka, suggesting that the disruption in the utilisation of ANC services has not recovered in most divisions by the end of 2021 (**Figure 3**).

### Monthly change: institutional delivery

**Figure 4:**
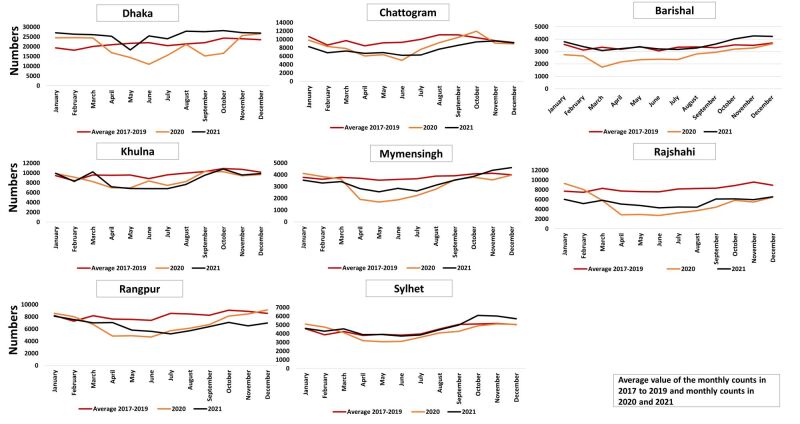
Monthly trend of institutional delivery using DHIS2 data set from 2017 to 2021. DHIS2 – District Health Information Software, version 2.

In the year 2020, there was a noticeable drop in April and rise in August (**Figure 4**). The highest decline observed in Dhaka in April 2020. In contrast, the year 2021 exhibited a significant rebound in the values. Also, the values for year 2021 were aligned with the values of average of 2017–2019 with a slight variation in all divisions. The utilisation of delivery services recovered by the end of 2021 in all division except Rangpur and Rajshahi.

### Monthly change: caesarean section

**Figure 5:**
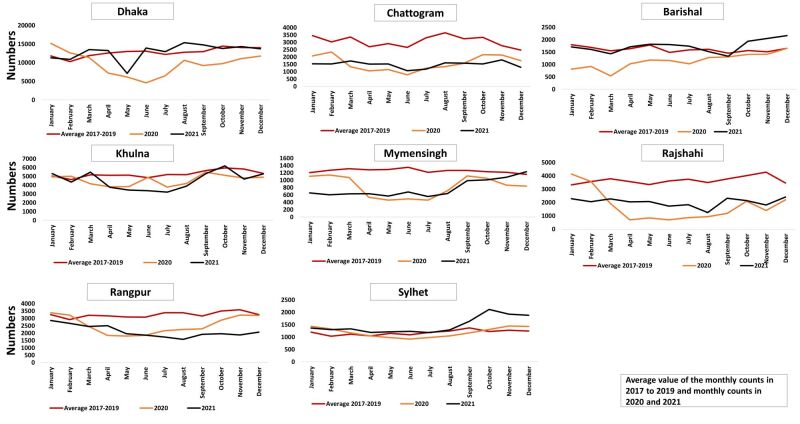
Monthly trend of caesarean section using DHIS2 data set from 2017 to 2021. DHIS2 – District Health Information Software, version 2.

The average values of caesarean section during the years 2017–2019 remained relatively stable in all divisions with slight monthly fluctuations (**Figure 5**). In the year 2020, there was a noticeable drop in April and simultaneous rise in August. The year 2021 exhibited a visible rebound in the values. The values for year 2021 were aligned with the values of average of 2017–2019 with a slight variation in all divisions. There was a drop-in caesarean section but it recovered in 2021 in Dhaka, Barishal, Khulna and Sylhet.

### Segmented regression: ANC first visit

The likelihoods of ANC first visit in these divisions during 2020 and 2021 were lower compared to the reference period 2017–2019 (**Table 1**). The number of ANC first visits was significantly less frequent during 2020–2021. The lowest value was observed in the Chattogram in 2020 (IRR = 0.66; 95% CI = 0.55–0.79) and Rajshahi in 2021 (IRR = 0.71; 95% CI = 0.60–0.82).

**Table 1:** Results from segmented regression on first antenatal care visit interrupted time series presented in incidence rate ratio with 95% confidence interval

Site	2020		2021	
	**IRR (CI)**	***P*-value**	**IRR (CI)**	***P*-value**
Bangladesh	0.84 (0.72–0.99)	0.042	0.82 (0.75–0.89)	<0.001
Dhaka	1.04 (0.85–1.28)	0.704	0.72 (0.62–0.84)	<0.001
Chattogram	0.66 (0.55–0.79)	<0.001	0.90 (0.83–0.98)	0.019
Barishal	0.73 (0.71–0.75)	<0.001	0.98 (0.85–1.15)	0.840
Khulna	0.82 (0.76–0.89)	<0.001	0.73 (0.68–0.79)	<0.001
Mymensingh	0.99 (0.98–1.02)	0.993	0.99 (0.94–1.06)	0.992
Rajshahi	0.67 (0.54–0.83)	<0.001	0.71 (0.60–0.82)	<0.001
Rangpur	0.70 (0.69–0.71)	<0.001	1.03 (0.92–1.15)	0.634
Sylhet	0.77 (0.66–0.91)	0.002	0.84 (0.77–0.92)	<0.001

CI – confidence interval, IRR – incidence rate ratio

### Segmented regression: institutional delivery

Overall, Bangladesh observed 20% decline in 2020 and 13% decline in 2021 in the number of institutional deliveries compared to pre-COVID period (**Table 2**). Barishal observed the highest decline in 2020 (IRR = 0.64; 95% confidence interval (CI) = 0.60–0.68) and Rajshahi in 2021 (IRR = 0.53; 95% CI = 0.50–0.57).

**Table 2:** Results from segmented regression on institutional delivery interrupted time series presented in incidence rate ratio with 95% confidence interval

Site	2020		2021	
	**IRR (CI)**	***P*-value**	**IRR (CI)**	***P*-value**
Bangladesh	0.80 (0.72–0.89)	<0.001	0.87 (0.83–0.91)	<0.001
Dhaka	0.79 (0.67–0.93)	0.004	1.04 (1.02–1.07)	0.001
Chattogram	1.00 (0.89–1.13)	0.945	0.88 (0.76–1.01)	0.073
Barishal	0.64 (0.60–0.68)	<0.001	0.96 (0.90–1.02)	0.165
Khulna	0.76 (0.70–0.82)	<0.001	0.83 (0.77–0.89)	<0.001
Mymensingh	0.73 (0.64–0.83)	<0.001	0.68 (0.62–0.75)	<0.001
Rajshahi	0.66 (0.55–0.81)	<0.001	0.53 (0.50–0.57)	<0.001
Rangpur	0.70 (0.59–0.82)	<0.001	0.88 (0.83–0.93)	<0.001
Sylhet	0.76 (0.70–0.83)	<0.001	0.74 (0.69–0.79)	<0.001

CI – confidence interval, IRR – incidence rate ratio

### Segmented regression: caesarean section

The likelihood of taking caesarean section in these divisions during 2020–2021 was lower compared to the reference period 2017–2019, 30% decline observed in 2020 and 25% in 2021 (**Table 3**). The lowest value was observed in the Barishal division (IRR = 0.48; 95% CI = 0.44–0.53) in 2020. In 2021 highest decline observed in Mymensingh (IRR = 0.37; 95% CI = 0.32–0.43).

**Table 3:** Results from segmented regression on caesarean section interrupted time series presented in incidence rate ratio with 95% confidence interval

Site	2020		2021	
	**IRR (CI)**	***P-*value**	**IRR (CI)**	***P-*value**
Bangladesh	0.70 (0.59–0.82)	<0.001	0.75 (0.72–0.79)	<0.001
Dhaka	0.74 (0.63–0.87)	<0.001	0.76 (0.74–0.79)	<0.001
Chattogram	0.90 (0.70–1.17)	0.448	0.90 (0.72–1.12)	0.349
Barishal	0.48 (0.44–0.53)	<0.001	0.99 (0.89–1.01)	0.929
Khulna	0.79 (0.75–0.84)	<0.001	0.77 (0.69–0.86)	<0.001
Mymensingh	0.70 (0.59–0.82)	<0.001	0.37 (0.32–0.43)	<0.001
Rajshahi	0.64 (0.48–0.85)	0.002	0.48 (0.45–0.52)	<0.001
Rangpur	0.77 (0.68–0.88)	<0.001	0.83 (0.78–0.89)	<0.001
Sylhet	0.74 (0.63–0.85)	<0.001	0.72 (0.63–0.81)	<0.001

CI – confidence interval, IRR – incidence rate ratio

## DISCUSSION

Our study investigated the divisional disparities of the indirect impact of COVID-19 on key indicators of maternal health in Bangladesh using routine health information data. Our findings suggest during the first lockdown in 2020, all three maternal health indicators declined. We also observed decline in utilisation in 2021 compared to pre-COVID years, but the highest decline was observed during 2020. The number of women delivering through caesarean section declined around one-third during COVID-19, which the most among all three indicators. We observed lower incidence rate for ANC first visit in Chattogram and Rajshahi division; institutional delivery in Barishal and Rajshahi division and caesarean section in Barishal and Mymensingh divisions after the onset of COVID-19 in Bangladesh.

Bangladesh's experience with maternal service utilisation during COVID-19 mirrors some aspects seen in other low- and middle-income countries but also presents unique challenges. Consistent with our findings, Wangmo and colleagues using national health information system of Bangladesh (DHIS2) found substantial reductions in the essential health services including maternal health services utilisation from April 2020 to May 2020 [27]. These services include the number of outpatient appointments, inpatient admissions, first ANC visits and institutional normal vaginal deliveries, as well as the percentage of children fully immunised by the Expanded Programme on Immunisation, assessed for each month and level of health facility. Other studies in Asia and Africa including countries such as Uganda, Pakistan, India, Kenya, Zambia, Demographic Republic of the Congo, Haiti, Liberia and South Africa have also found declines in maternal service utilisation during the first of months of the pandemic [15,28–31]. Most public health facilities in Bangladesh were over-crowded and reported to have low coverage and quality of services, poor hygiene and sanitation practises during pandemic [32,33]. During COVID-19 pandemic, care seekers faced many challenges. There were socio cultural reasons for this decline in the service utilisation during COVID-19. The plausible reason for decrease in selected maternal health services in Bangladesh could be a switched focus towards pandemic, decline in economy, the execution of area-wise lockdown, travel restrictions, shortened visiting hour by government in the public facilities, lack of medical equipment such as personal protective equipment for health workers [34–36]. Due to COVID-19 pandemic, most of the health workers’ duty was redesigned to serve the emergency response which led to human resource scarcity in the public facilities [36]. Concerns for safety issues and fear of having COVID-19 infection in both the patients and health workers contributed to the decline in the utilisation of selected maternal health services [37]. Fear of contracting the virus led many pregnant women to avoid hospital visits, resulting in reduced access to essential maternal health services [15]. In Bangladesh, other factors included the unavailability of transport during the pandemic [38]. However, Bangladesh implemented innovative solutions like midwife-led telemedicine services to address some of these gaps. This contrasts with countries such as Nigeria and South Africa, where similar declines occurred but without widespread telemedicine initiatives [15].

Our observation of declining utilisation in 2021 compared to pre-COVID years with highest decline during 2020 is similar to the study of Angeles and colleagues [28]. Reductions in ANC first visit and institutional deliveries during first month of pandemic with a gradual and incomplete recovery by August 2020 have been shown on national level in Bangladesh [28]. Service utilisation increased gradually when the lockdown was lifted, but did not entirely bounce back to pre-pandemic levels [39]. Limitation on public transit and scarcity of transportation was being existed from March 2020–2021 in Bangladesh that could be a feasible cause of reduced maternal health service utilisation [38]. However, significant increase in trends were noticed by Ebola post-outbreak in Liberia which took almost one year to regain than the pre-outbreak level [40]. Recent study using routine monitoring data in Pakistan found a drop of 57% in caesarean section rate and 37% in institutional delivery during January–May 2020 and an increase in June–September 2020 with another decline in October–December 2020 [37]. Another study in the Demographic Republic of the Congo reported decreased ANC first visit after the pandemic started but institutional delivery remained unaffected. Antenatal care service utilisation recovered when the lockdown was lifted, but the trend in rates of maternal health services (ANC first visit and institutional delivery) did not rise over time as compared to the trend of expected pre-COVID-19 situation [39]. The COVID-19 pandemic resulted in serious disruption in ANC and institutional delivery services for one month in Cameroon, Demographic Republic of the Congo, Liberia, Malawi, Mali, Nigeria, Sierra Leone and Somalia with a different size and time frame [41]. Research in South Africa highlighted a disproportionate impact of COVID-19 pandemic on ANC first visit and caesarean section in public facilities in different divisions from April 2020 to June 2021 [31]. Another study in Bangladesh noticed a gradual but not parallel increase in service utilisation during 2021 which supports our findings [27]. This may be due to a flexible lockdown which expected to simultaneously trade off both public health and economic restart, possibly to improve future life and to reopen the economy before the successful development of vaccines. This was put in place following the second wave of COVID-19 pandemic [42].

Our observation highlighted divisional disparities in the selected maternal health services utilisation during and post-pandemic situation. Lower incidence rate was observed for ANC first visit in Chattogram and Rajshahi division; institutional delivery in Barishal and Rajshahi division and caesarean section in Barishal and Mymensingh divisions in Bangladesh. Studies have shown that women in Rajshahi division were less likely to have ANC visits during the pre-pandemic years compared to other divisions in Bangladesh [43,44]. In 2016, Sarker and colleagues, shared their field level experience in three districts of Rajshahi division and assumed that the socio-cultural factors are linked to delayed ANC services in the study areas [45]. Around 32% of the health workers were repurposed for the COVID-19 emergency response in Mymensingh division [27]. This may have generated a gap of human resource which may have caused lower maternal health services utilisation during 2020–2021 in Mymensingh [27].

A major drawback of DHIS2 in Bangladesh is its inability to include data from private health facilities. This omission leads to incomplete health information, which can impede effective health planning and policy-making. Our data showed a declining trend of number of first ANC visit which is contradictory from the survey data [46]. One of the potential reasons for this could be the transition from public to private facilities for seeking ANC. As DHIS2 lacks private health facility data, we observed a decline in the overall ANC trend.

We also acknowledge the data quality may have been an issue while using and interpreting DHIS2 data, especially in the case of ANC information in this analysis. A low resource setting with technical infrastructure, lack of skilled health worker, insufficient software development and data security issues may have created challenges in maintaining the electronic data system in Bangladesh [47]. Lack of training on data management regarding HMIS dashboard, insufficient supervision from district health manager and higher authorities, absence of feedback on the quality of monthly reports influenced the data quality [48,49]. Gaps in data availability, under-reporting, incompleteness and inconsistency of the reports were also evident in the DHIS2 data in Nigeria [50,51]. Inadequate human resource, lack of standard operating procedure contributed to poor data reporting [48]. Follow up patients are preferably enrolled as new patients by health care providers that raises data quality issue of inaccurate number of service utilisation [16]. In some instances, data from peripheral level health facilities takes an average of three months to reach the central office. Patient load also affected the online registration and data entry process. Lack of human resource, slow internet connectivity, frequent changes in DHIS2 versions, maintaining both manual registry and electronic database were identified as barriers to maintain quality routine data in DHIS2 in Bangladesh [16]. Checking quality and providing feedback on a regular basis from higher authority helps to reduce data errors in DHIS2 [16].

### Policy and programme implications

There are many initiatives taken by government, private (Non-Governmental Organizations (NGOs) and International Non-Governmental Organizations (INGOs)) organisations during COVID-19 [34,52,53]. The Government of Bangladesh initiated several schemes, interventions, policies to continued uninterrupted health services across the country, but the care seeking did not adequately improve after COVID-19 in the immediate next year according to the data on public facilities of Bangladesh. In order to act in timely manner Bangladesh, need to have early strategic planning for combating evolving challenges during pandemic. Appropriate response plans and management are crucial for the sustainability of the nation. An elaborate national plan with focus on divisional differences may help government in effective planning and resource optimisation. Special focus should be given to Chattogram, Rajshahi, Barishal, and Mymensingh for future resource planning.

### Strengths and limitations

This study has several strengths that further contribute to the potential of data in DHIS2 for local, real-time monitoring of selected maternal health services. The inclusion of the large sample size of DHIS2 data from different divisions in Bangladesh allowed for conclusions to be drawn across regions. The unique data allowed for exploration of both yearly and monthly trends using interrupted time series data approach to understand seasonal patterns and time periods, and to see the variation in the number of maternal health services utilisation which were mostly disrupted by COVID-19 period.

There are limitations in estimating the coverage of maternal health services through the DHIS2 in Bangladesh. First, we observed a wide-spread absence of population data, decreased quality and completeness of data collection during the COVID-19 pandemic. A significant reason for incomplete data in the national routine health information system in Bangladesh could be uncovered data of private facilities under the DHIS2 network [16]. Therefore, the true impact on the coverage of maternal health services might be less than what was originally observed based on DHIS2 utilisation data in the early phase of the pandemic.

Second, there were gaps, delays in the documentation and reporting of routine services and lack of control in DHIS2 electronic version in the initial phase of the pandemic, particularly during the lockdown period. The quantitative analysis of the changes in the level of service utilisation is prone to bias from several issues of HMIS data. Third, absence of data on maternal and neonatal health services from Directorate General of Family Planning and private facilities in the DHIS2 dashboard contributes to a gap in generalisability regarding disparities in geographical and periodic findings [16,22,54]. Moreover, the lack of varied independent variables and qualitative data limits a thorough understanding of service recipient characteristics. Third, the established formula for adjusting the incomplete reporting does not contain the validity parameter of the estimate. Therefore, it was difficult for us to ascertain how the choices of adjustment factors affected the validity of the results. Fourthly, we have used the facility-based data and could not explore the socio-cultural context. More detailed analysis on how socio-cultural factors influence maternal health service utilisation in Bangladesh could strengthen our analysis. Lastly, we did not identify adequate evidence to explain the regional disparities in maternal service utilisation during COVID-19. We discussed the disparities, but further research endeavours will be required to better explain the reasons behind these differences.

## CONCLUSIONS

Our analysis sheds light on disruptions in selected maternal health services following COVID-19 and afterward in Bangladesh. Regional differences in maternal health service utilisation during and after COVID-19 were apparent, particularly in Chattogram, Rajshahi, Barishal, and Mymensingh, where the effects were most pronounced. This will inform the policy makers for timely response during future pandemics considering divisional disparities. The analytical approach used in this paper could also be utilised as a form of surveillance to detect service interruptions beyond the pandemic in Bangladesh.

## Additional material


Online Supplementary Document
