# Supplementary material for: Impact of COVID-19 on the utilisation of maternal health services in Bangladesh: A division-level analysis
Source: J Glob Health. 2024 Nov 22;14:05040. doi: 10.7189/jogh.14.05040 (PMC11583111; doi:10.7189/jogh.14.05040)
Supplement: Online Supplementary Document [file jogh-14-05040-s001.pdf]

Supplementary materials:

Figure S1: Monthly drop for maternal health indicators by eight divisions in Bangladesh

ANC 1st visit:

Dhaka

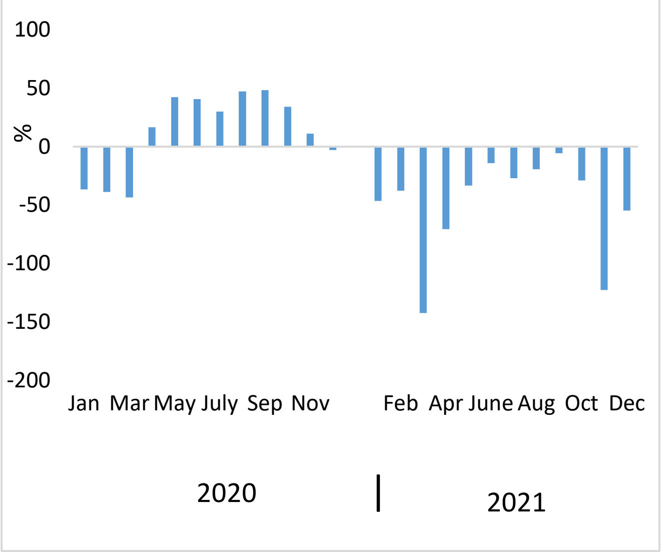

Chattogram

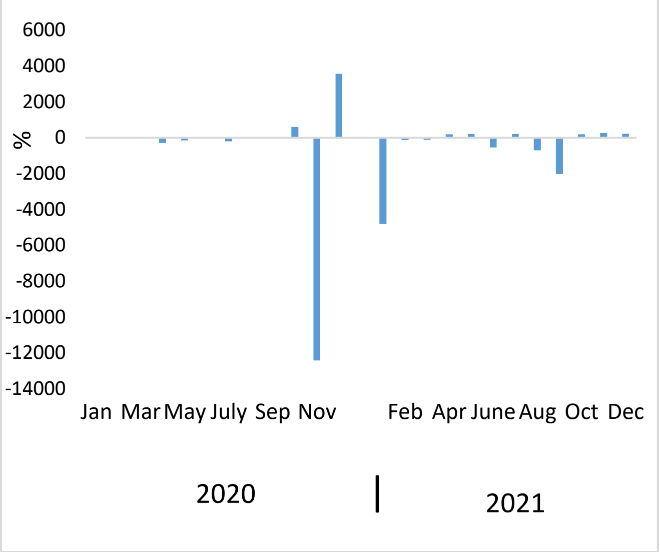

Barishal

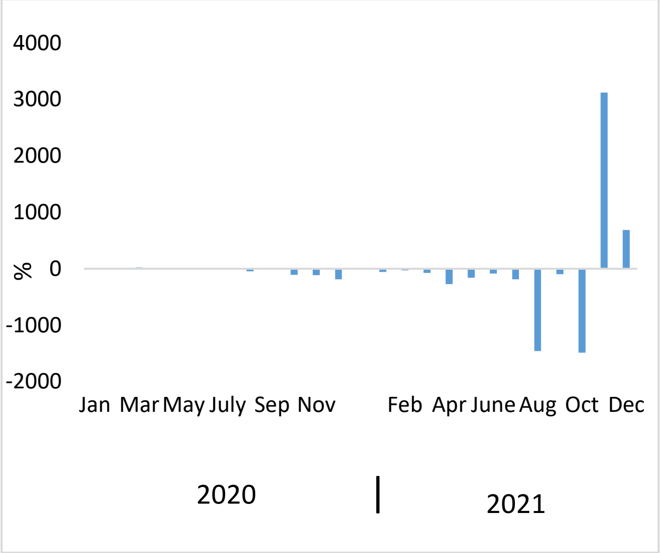

Khulna

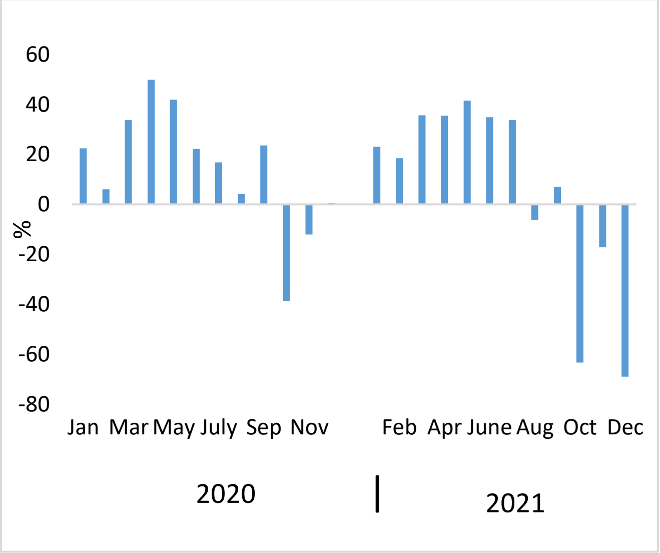

Mymensingh

Rajshahi

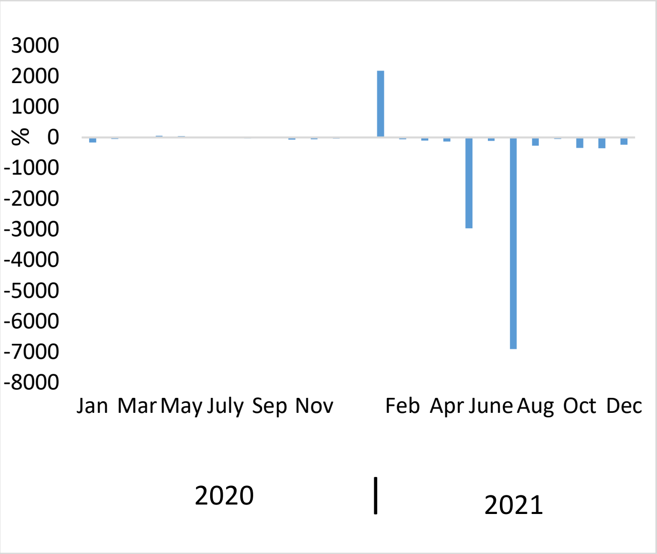

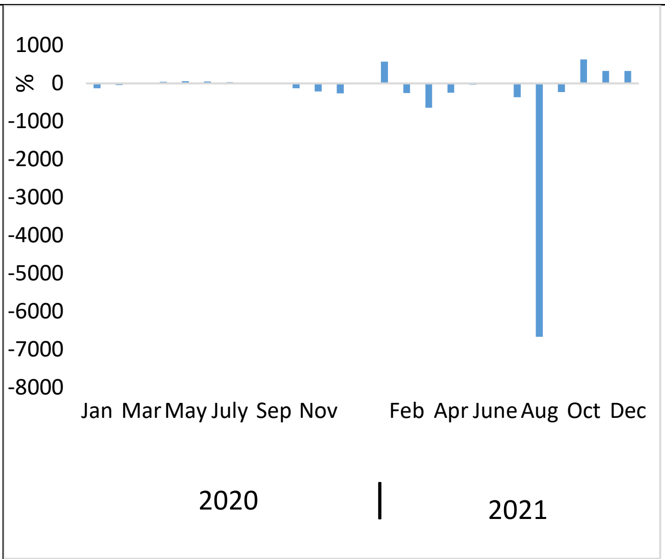

Rangpur	Sylhet

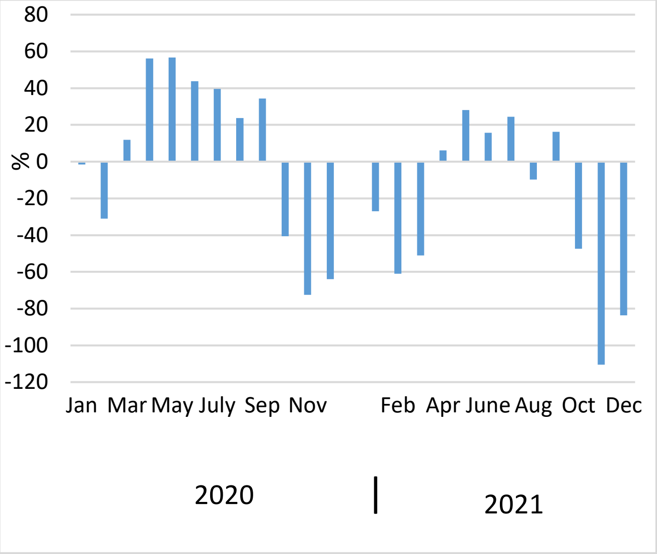

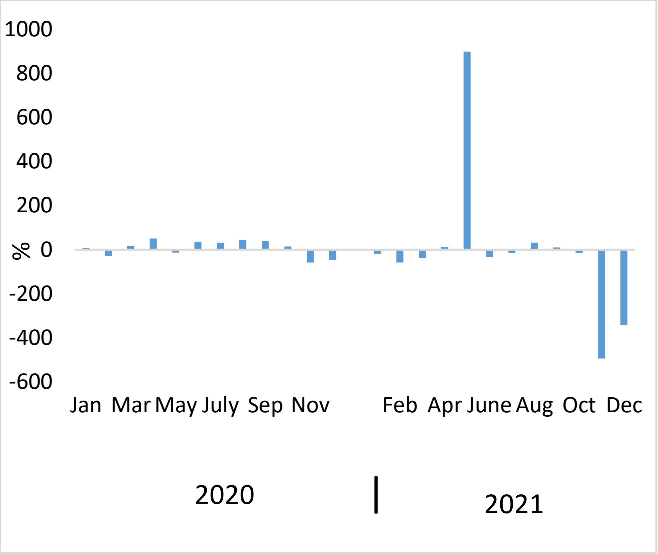

Institutional Delivery:

Dhaka	Chattogram

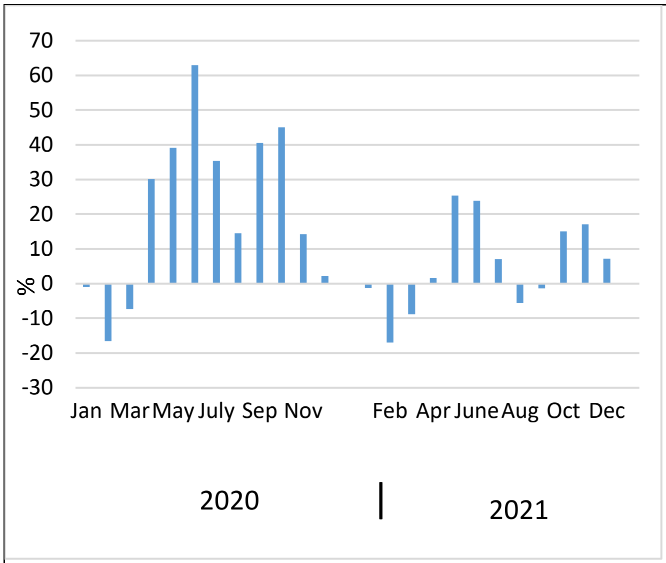

Barishal

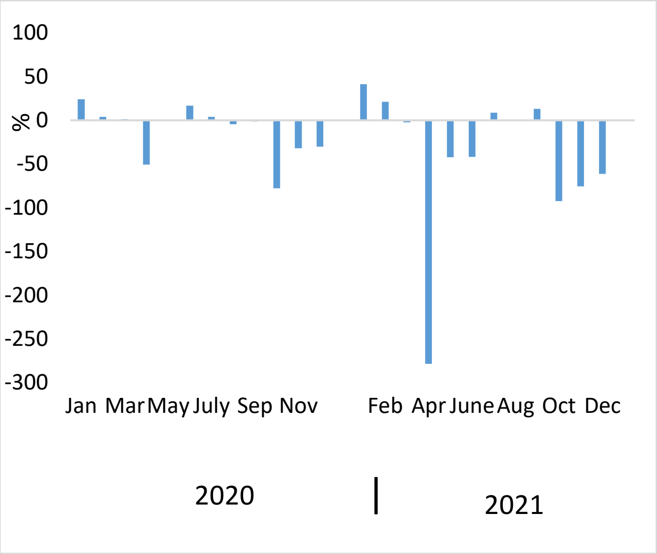

Khulna

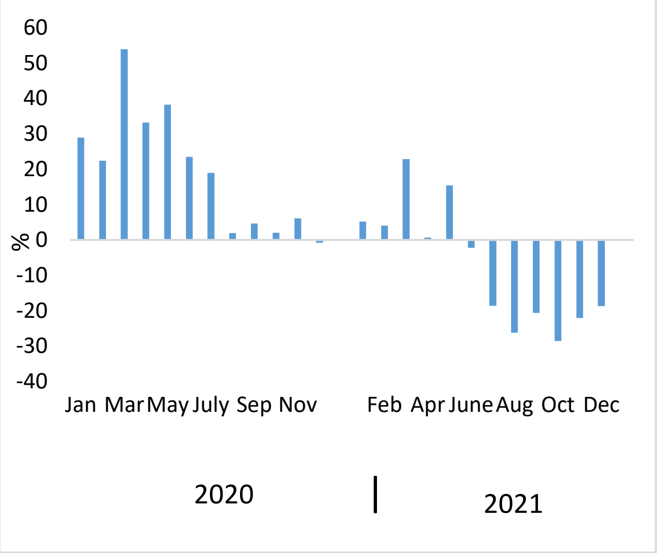

Mymensingh

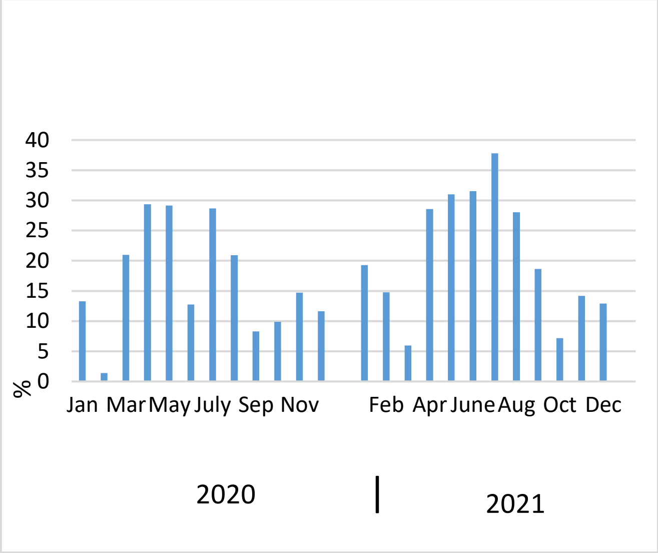

Rajshahi

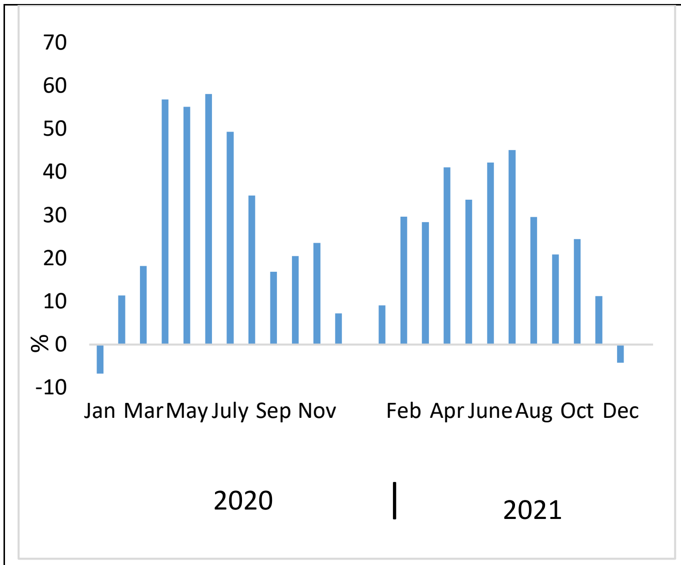

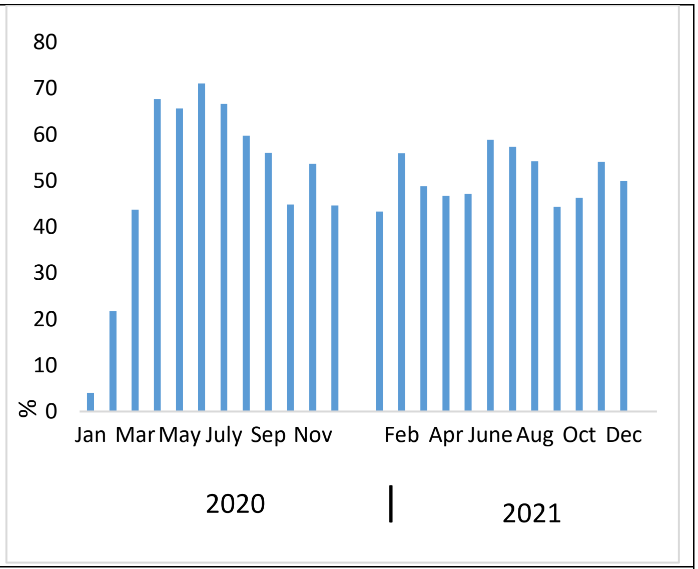

Rangpur	Sylhet

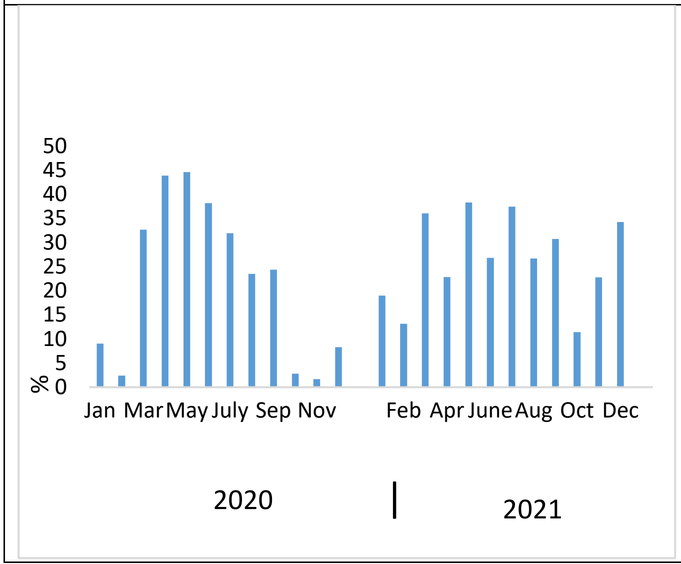

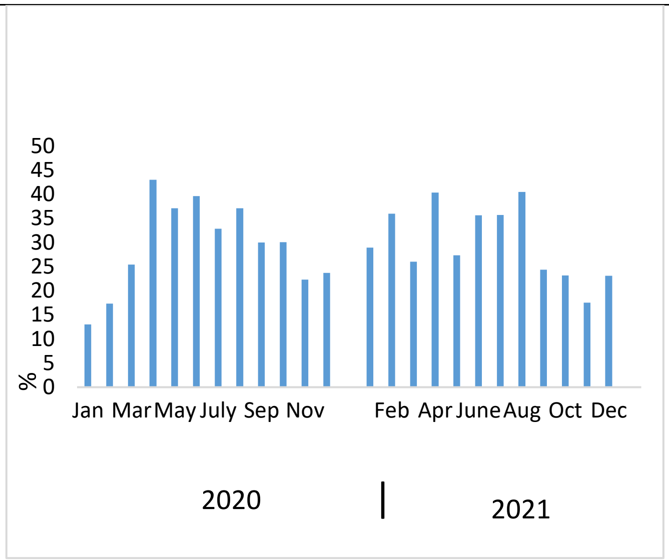

Caesarean section:

Dhaka

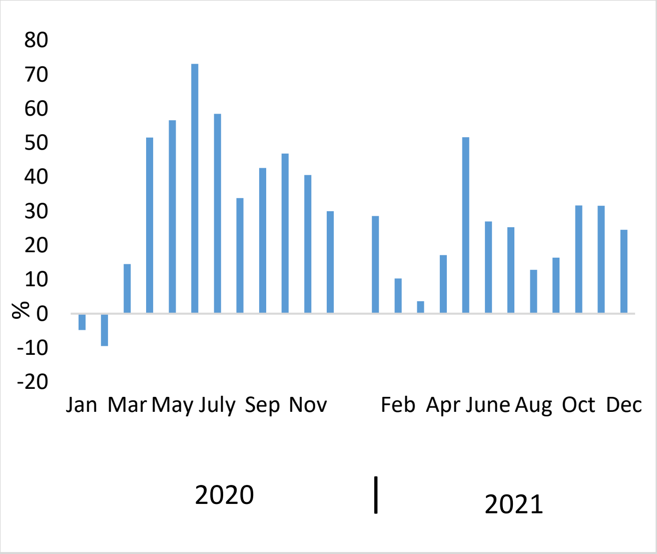

Chattogram

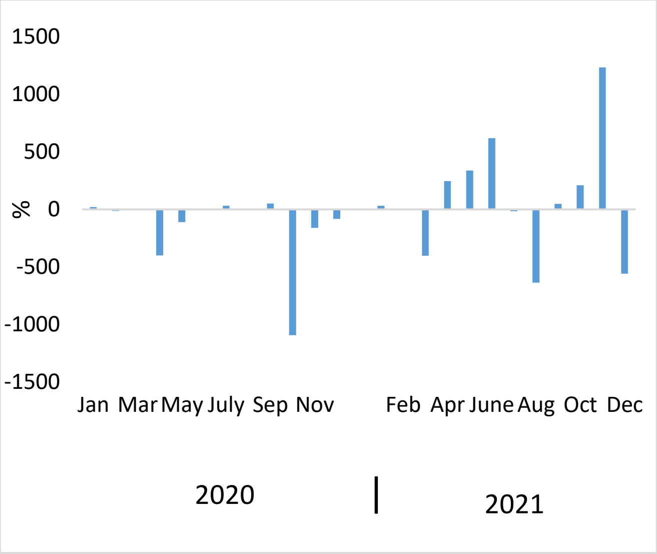

Barishal

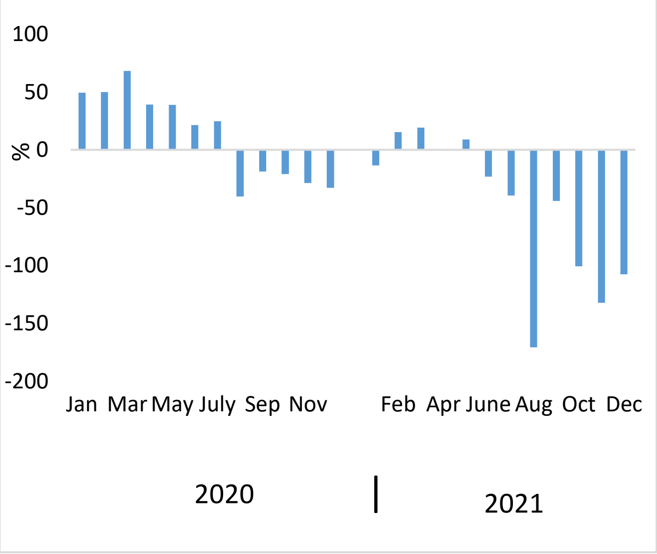

Khulna

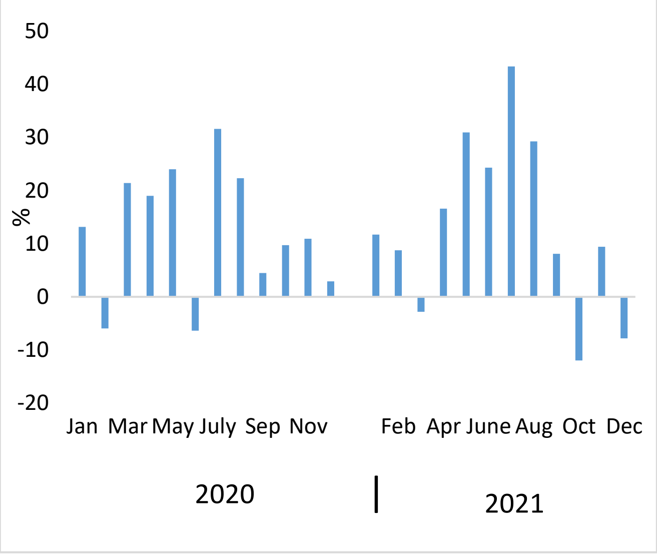

Mymensingh

Rajshahi

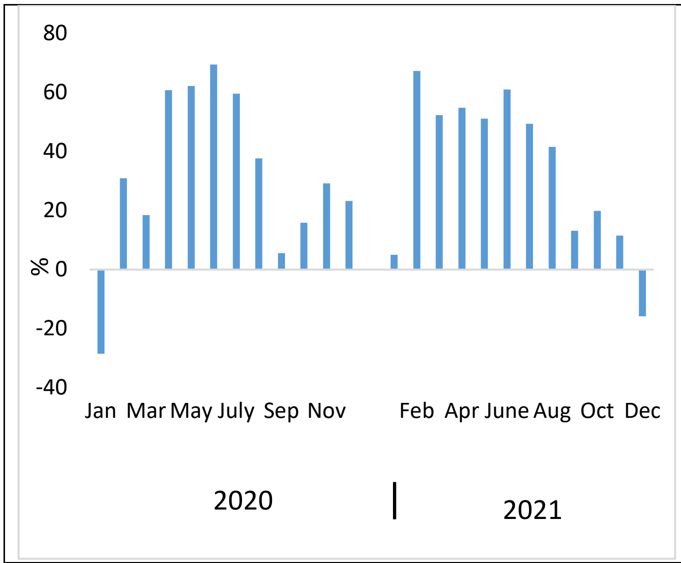

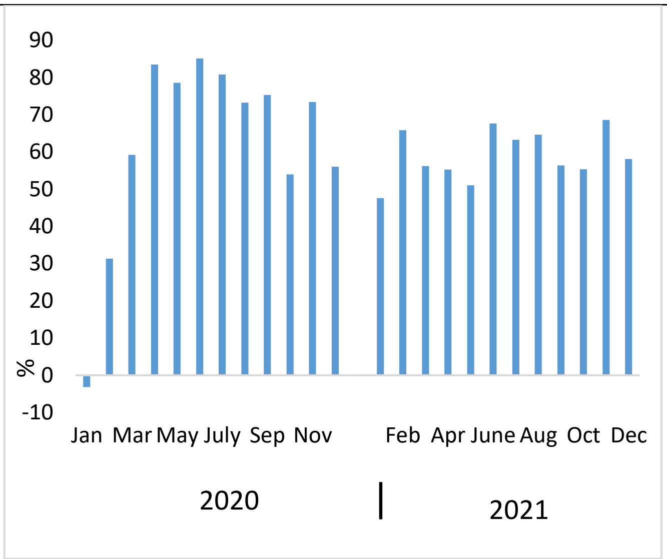

Rangpur	Sylhet

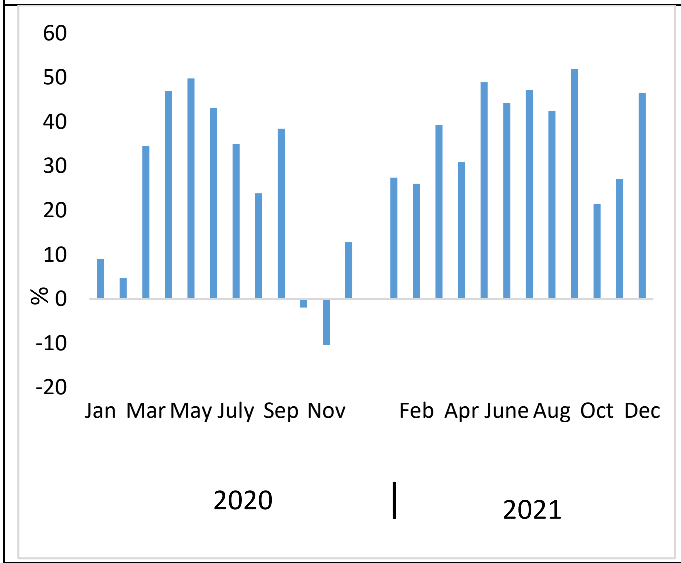

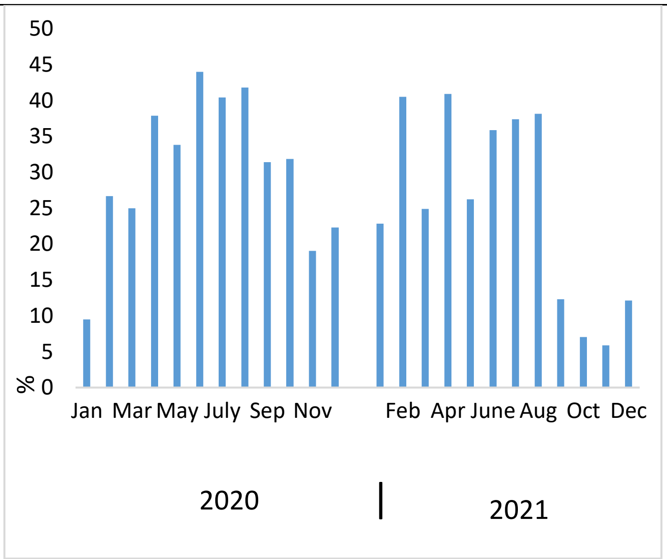

Appendix S1:

Data quality of DHIS2:

Data quality can be assessed by completeness and timeliness of facility reporting. DHIS2 provides information on the completeness and timeliness of collected reports. Maternal health indicators are included in the Emergency Obstetric and Newborn Care (EmONC) report. The completeness of a specific report/data set, such as the reported EmONC, in the DHIS2 database was evaluated by comparing the total number of reported health facilities/community workers in the DHIS2 database with the total number expected to report. As of December 2021, the data completeness rate for EmONC in DHIS2 was below 90%, indicating that the quality of DHIS2 data for maternal health indicators is poor and incomplete. Reporting timeliness in DHIS2 was assessed based on the availability of reports within a fixed reporting date. The timeliness of

EmONC reports was alarmingly low, falling below 60% during our study period. We have added this information in the supplementary material for better transparency.

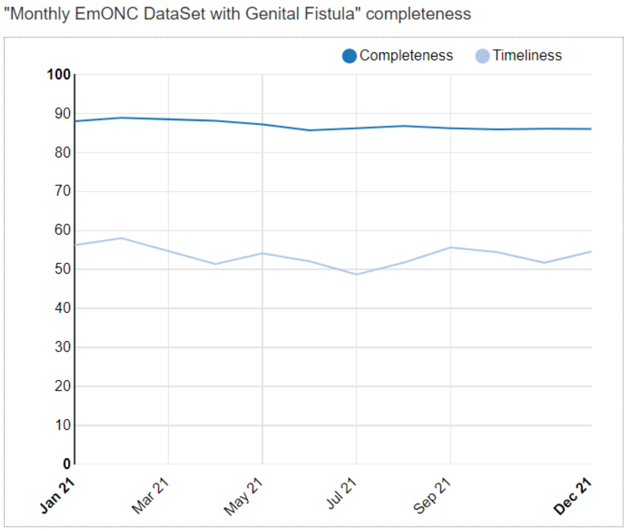

Appendix S2:

Seasonality adjustment of the segmented regression model

The general form of segmented regression,

$$Y_t = \beta_0 + \beta_1 * time_t + \beta_2 intervention_t + \beta_3 * time\ after\ intervention_t + e_t$$

Where, Y_t is the mean number of deaths in month *t*; time is a continuous variable indicating time in months at time *t* from the start of the observation period; intervention (any program or event happened like COVID-19 pandemic) is an indicator for time *t* occurring before (intervention $= 0$) or after (intervention $= 1$) the covid-19 pandemic; and time after intervention is a continuous variable counting the number of months after the intervention at time *t*, coded 0 before the covid-19 pandemic and (1 to continue) after the covid-19 pandemic.

The seasonal pattern is an additional term in the data that repeats every 12 months [1]. According to the Fourier series expansion theorem, any repeating signal with a time period *T* can be represented as a summation of sine and cosine functions. For monthly data, where *T* = 12, we used Fourier series to fit the seasonality component, that is, linear combinations of sine and cosine functions. For example, to model monthly counts, the model can be written as follows [2],

$$Y_t = \beta_0 + \beta_1 * time_t + \beta_2 intervention_t + \beta_3 * time\ after\ intervention_t + \beta_s * \sin(\frac{2\pi t}{T}) + \beta_c * cos(\frac{2\pi t}{T}) + e_t$$

References:

1. Bramness JG, Walby FA, Morken G, Røislien J. Analyzing seasonal variations in suicide with Fourier Poisson time-series regression: a registry-based study from Norway, 1969–2007. American journal of epidemiology. 2015;182(3):244-54.
2. Ramanathan K, Thenmozhi M, George S, Anandan S, Veeraraghavan B, Naumova EN, Jeyaseelan L. Assessing seasonality variation with harmonic regression: accommodations for sharp peaks. International journal of environmental research and public health. 2020;17(4):1318.
